# Profiling postgraduate workplace-based assessment implementation in Ireland: a retrospective cohort study

**DOI:** 10.1186/s40064-016-1748-x

**Published:** 2016-02-20

**Authors:** Aileen Barrett, Rose Galvin, Yvonne Steinert, Albert Scherpbier, Ann O’Shaughnessy, Gillian Walsh, Mary Horgan

**Affiliations:** Education and Professional Development Unit, Royal College of Physicians of Ireland, Frederick House, 19 South Frederick St, Dublin 2, Ireland; School of Medicine, College of Medicine and Health Sciences, Brookfield Health Sciences Complex, University College Cork, Cork, Ireland; Discipline of Physiotherapy, Department of Clinical Therapies, Faculty of Education and Health Sciences, University of Limerick, Limerick, Ireland; Centre for Medical Education, Faculty of Medicine, McGill University, Lady Meredith House, 1110 Pine Avenue West, Montreal, QC H3A 1A3 Canada; Faculty of Health, Medicine and Life Sciences, University of Maastricht, Universiteitssingel 60, 6229 ER Maastricht, The Netherlands

**Keywords:** Workplace-based assessment, Postgraduate medical education, Retrospective cohort study

## Abstract

In 2010, workplace-based assessment (WBA) was formally integrated as a method of formative trainee assessment into 29 basic and higher specialist medical training (BST/HST) programmes in six postgraduate training bodies in Ireland. The aim of this study is to explore how WBA is being implemented and to examine if WBA is being used formatively as originally intended. A retrospective cohort study was conducted and approved by the institution’s Research Ethics Committee. A profile of WBA requirements was obtained from 29 training programme curricula. A data extraction tool was developed to extract anonymous data, including written feedback and timing of assessments, from Year 1 and 2 trainee ePortfolios in 2012–2013. Data were independently quality assessed and compared to the reference standard number of assessments mandated annually where relevant. All 29 training programmes mandated the inclusion of at least one case-based discussion (max = 5; range 1–5). All except two non-clinical programmes (93 %) required at least two mini-Clinical Evaluation Exercise assessments per year and Direct Observation of Procedural Skills assessments were mandated in 27 training programmes over the course of the programme. WBA data were extracted from 50 % of randomly selected BST ePortfolios in four programmes (n = 142) and 70 % of HST ePortfolios (n = 115) in 21 programmes registered for 2012–2013. Four programmes did not have an eligible trainee for that academic year. In total, 1142 WBAs were analysed. A total of 164 trainees (63.8 %) had completed at least one WBA. The average number of WBAs completed by HST trainees was 7.75 (SD 5.8; 95 % CI 6.5–8.9; range 1–34). BST trainees completed an average of 6.1 assessments (SD 9.3; 95 % CI 4.01–8.19; range 1–76). Feedback—of varied length and quality—was provided on 44.9 % of assessments. The majority of WBAs were completed in the second half of the year. There is significant heterogeneity with respect to the frequency and quality of feedback provided during WBAs. The completion of WBAs later in the year may limit available time for feedback, performance improvement and re-evaluation. This study sets the scene for further work to explore the value of formative assessment in postgraduate medical education.

## Background

Workplace-based assessment (WBA) was originally mooted as a formative—or ‘assessment-*for*-learning’— practice with a primary aim of impacting trainee learning and development and to assist in focusing the trainee’s learning plans (Norcini et al. 1995). The format of the assessment takes place in real time, with the supervisor observing the trainee in a specific aspect of clinical practice. Since its introduction many tools have been developed (Kogan et al. 2009) to structure feedback on specific aspects of a trainee’s performance.

Over time, the use of WBA has expanded to include a quality assurance role (Black and Welch 2009) and has been mooted as a method of early identification of poor performance (Cohen et al. 2009). Implementation of WBA internationally has met with varied levels of success and acceptability (Fokkema et al. 2013) with many ongoing reservations regarding the practical feasibility of performing multiple assessments in order to comply with recommendations for good reliability while attempting to maintain the formative function of these assessments (Bok et al. 2013). The introduction of what is viewed as an additional demand on trainer and trainee time, in an increasingly busy and unstructured environment has also impacted on the acceptability of these learning ‘innovations’ (Fokkema et al. 2013; Fokkema and Teunissen 2013).

One of the main criticisms of the implementation of WBA has emerged where the assessments are not mapped to training programme outcomes or aligned with a defined programme of assessment throughout training (Driessen and Scheele 2013). Poor communication of the formative purpose of WBA has also emerged as a critical barrier to successful implementation of these tools (Bok et al. 2013). Attempts to communicate the formative nature of the assessments in the UK by changing the name to ‘supervised learning events’ have also been met with mixed opinions (Ali 2014).

The focus of workplace-based assessment research has, however, begun to take a new direction. While acknowledging the limitations of workplace-based assessment as individual summative judgments of performance, the place of these tools within a programme of assessment hinges more on their validity as formative assessments, than their reliability as summative assessments (Cook et al. 2014; Hatala et al. 2015; Cook et al. 2015; St-Onge and Young 2015). The role of narrative feedback in this conceptualisation of validity becomes therefore increasingly important.

In the Irish context, WBA was introduced as mandatory component of postgraduate medical training across six training bodies in 2010. The mini-clinical evaluation exercise (Mini-CEX) and case-based discussion (CbD) were included across all disciplines while the Direct Observation of Procedural Skills (DOPS) assessment was included for disciplines with procedural skill requirements. The Objective Structured Assessment of Technical Skills (OSATS)—with procedure-specific adaptations—was implemented in both basic and higher specialist training programmes in Obstetrics and Gynaecology. Procedure-specific DOPS forms were also developed and implemented for higher specialist training in gastroenterology.

### Research aim

The research question posed by this study is ‘how have workplace-based assessments been integrated into higher specialist training programmes in medicine in Ireland?’

The study comprises three key objectives:

1. to describe the level of implementation of WBA in postgraduate Basic Specialist Training (BST) and Higher Specialist Training (HST) programmes in one postgraduate medical training institution in Ireland.
2. to compare the findings with those published from other training jurisdictions.
3. to explore the quality of written feedback provided in these assessments.

### Conceptual framework

This study was guided by work in two key areas of educational research, formative assessment theory (Clark 2012; Bennett 2011) and guidelines for good practice in effective feedback (Nicol and Macfarlane-Dick 2006; Watling 2014). Contemporary formative assessment theory proposes that all assessment should guide learning and development (Bok et al. 2013, 2015). Guidelines for good practice suggest that in order to be effective, feedback must be, among other factors, specific, timely and result in a further plan for development (Nicol and Macfarlane-Dick 2006). The mechanisms by which feedback can be deemed to be successful in this purpose remain challenging to elucidate and the learner’s response to that feedback—and therefore its ultimate use—is less predictable (Watling et al. 2012, 2013a, b). This study therefore only addressed evidence of feedback provided on written assessments and did not attempt to link this directly to evidence of learning.

## Methods

### Study design

This study was conducted using a retrospective cohort design. The STROBE standardised reporting guidelines were followed to ensure the standardised conduct and reporting of the research (Vandenbroucke et al. 2007; von Elm et al. 2007). Ethical approval was obtained from the institution’s Research Ethics Committee.

### Setting and study size

The study was conducted over a 3-month period from September to December 2013. Data were extracted anonymously from trainee ePortfolios for the academic year 2012–2013 (July–July). In 2011 a new ePortfolio replaced an existing paper-based recording system for trainees commencing programmes in that year. Therefore only data for Year I and Year II trainees (BST and HST) were available to access for this study. In order to obtain a truly representative picture of the level of implementation of WBAs, and considering the small total population size, 50 % of registered BST ePortfolios and 70 % of HST ePortfolios were included in the study.

### Data extraction

**Fig. 1:**
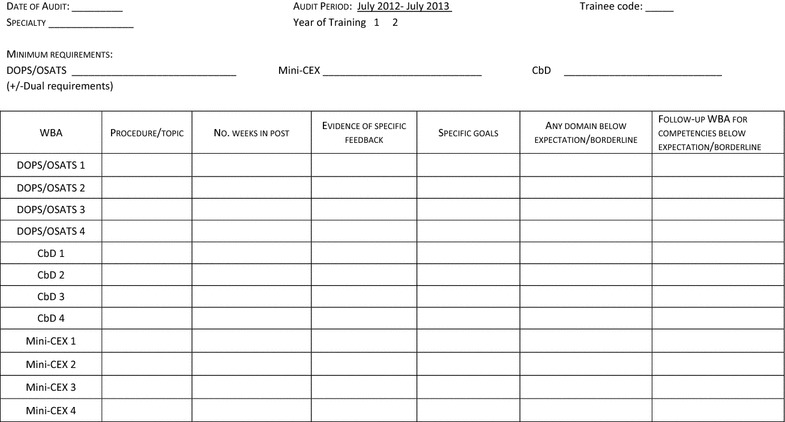
Data extraction tool

A data extraction tool was developed to extract anonymous data from trainee ePortfolios prior to the study commencement. This tool (Fig. 1) was designed to extract data on key ‘quality indicators’ of effective feedback, adapted from a number of sources including Nicol and MacFarlane-Dick’s ‘seven principles of good feedback practice’ (Nicol and Macfarlane-Dick 2006) and the WBA form content in use on these assessments. These indicators were assessed as binary outcomes (present/absent) and included the presence of learner-centred feedback specific to the assessment, learning goals and further follow-up where any competence was deemed to be ‘borderline’ or ‘below expectation’. The tool was piloted using data from five sample ePortfolios with one minor change to the use of ‘weeks’ instead of months in ascertaining the timing of the assessment completion. The timing of WBAs was therefore measured in weeks from the start of the academic year (9th July 2012).

### Quality check

Data were extracted by the principal investigator (AB) and a quality check of 10 % of the data extraction sheets was conducted by a second author (RG) prior to analysis. No extraction errors were identified; however it was agreed by the two authors to exclude three trainees’ data from the final analysis due to completion errors identified in those ePortfolios.

### Data analysis

The profile of WBA requirements was analysed descriptively from an Excel spreadsheet as were data extracted from ePortfolios. Binary data is presented as proportions where the denominator represents the total number of assessments completed in the programme. Summary means and standard deviations (SDs) are reported for continuous data, with corresponding 95 % CIs. Ranges are reported to illustrate the spread in the data. Data were compared to the reference standard number of assessments mandated annually, where relevant.

## Results

Data were extracted from a random selection of 50 % of BST ePortfolios in four programmes (n = 142) and 70 % of HST ePortfolios (n = 115) in 21 programmes registered for 2012–2013. Four programmes did not have an eligible trainee for that academic year. A total of 1142 individual assessments were analysed across both training programmes.

### WBA programme integration profile

All 29 programme curricula mandated at least one CbD annually (range 1–5). Annual mini-CEX assessments were required in all but two non-clinical specialties (range 1–4). DOPS requirements varied from 0 to 37 and most were required over the course of the training programme to allow for variations in opportunities to develop procedural skills in individual rotations. Two ‘non-procedural’ programmes did not have any DOPS requirement.

In HST, General Internal Medicine (GIM) training is completed alongside one of eight subspecialties. Trainees in these programmes complete at least 1 year of ‘high intensity GIM’ in which they must complete GIM curriculum requirements only and a ‘non-GIM’ year in which they complete their specialty requirements. For all other years, trainees complete requirements for both their GIM and specialty curriculum.

### WBA completion profile

The majority of trainee ePortfolios (164; 63.8 %) contained at least one completed WBA (76.5 % HST; 53.5 % BST). The average number of WBAs completed by individual HST trainees was 7.75 (SD 5.8; 95 % CI 6.5–8.9; range 1–34). BST trainees completed an average of 6.1 assessments (SD 9.3; 95 % CI 4.01–8.19; range 1–76).

The ‘quality indicators’ for each WBA are detailed in Tables 1 and 2.

**Table 1:** Basic specialist training results

	DOPS/OSATS	Mini-CEX	CbD
Total no completed	281	88	94
Average no. weeks in post before WBA completed	31.7 (95 % CI 30.1–33.3; SD 13.6; range 3–52)	35.2 (95 % CI 32.4–38.1; SD 13.5; range 7–52)	34.4 (95 % CI 31.4–37.3; SD 14.5; range 5–52)
Entries demonstrating defined goals	0	1 1.13 %	0
Entries with evidence of feedback	174 61.9 %	54 (61.3 %)	33 (35.1 %)
Evidence of any competence ‘borderline’ or ‘below expectation’	38 (13.5 %)	1 1.13 %	1 1.06 %
Evidence of follow-up	17 (44.7 %)	0	0

**Table 2:** Higher specialist training results

	DOPS/OSATS	Mini-CEX	CbD
Total no completed	359	153	167
Average no. weeks in post before WBA completed	30.1 (95 % CI 28.6–31.5; SD 14.1; range 1–52)	33.6 (95 % CI: 31.6–35.6′ SD 13.7; range 3–52)	32.6 (95 % CI: 31.7–35.5; SD 12.4; range 2–52)
Entries demonstrating defined goals	1 0.27 %	1 0.65 %	1 0.59 %
Entries with evidence of feedback	104 (28.9 %)	102 (66.6 %)	46 (27.5 %)
Evidence of any competence ‘borderline’ or ‘below expectation’	10 (2.78 %)	0	2 (1.19 %)
Evidence of follow-up	0	n/a	0

Assessments were mostly completed in the second half of the training year, after week 30.

Trainees were more likely to complete DOPS/OSATS than Mini-CEX or CbD assessments (ratio 3:1); 76 BST trainees completed 281 DOPS/OSATS, 88 Mini-CEX and 94 CbD assessments. A similar pattern emerged at HST where 88 trainees completed 359 DOPS/OSATS, 153 Mini-CEX and 167 CbD assessments. There were many errors in ePortfolio completion among ‘dual’ specialty trainees with WBAs entered into the incorrect logbook or use of the same WBA in both.

Feedback was provided on 44.9 % of assessments however the content of this feedback varied from one word (e.g. excellent) to complete sentences about the assessment episode. Trainer comments that pertained to the case (e.g. ‘complex case’) were not included as feedback in the analysis.

A total of 40 BST WBAs (8.63 %) and 12 HST WBAs (1.76 %) extracted contained a competence or component that was ‘borderline’ or ‘below expectation’. Of the 38 BST DOPS/OSATS assessments with a component deemed to be ‘borderline’ or ‘below expectation’, all were from within one speciality and 17 (44.7 %) were followed up with a second WBA in the same procedure. The 10 HST DOPS identified as ‘borderline’ or ‘below expectation’ were also from the same specialty; however none of these ePortfolios demonstrated evidence of follow-up.

## Discussion

The aim of this study was to determine the patterns of workplace-based assessment integration throughout postgraduate medical training curricula in six training bodies. Our main findings demonstrate that while the level of implementation has been varied, the majority of trainees have experienced at least one WBA during the academic year.

The picture that has emerged in this observational study compares in many ways with the issues identified internationally; particularly those related to ineffective feedback and limited formative impact. We identified that the *documentation* of effective written feedback was limited; however, as these assessments take place in real-time with the trainer and trainee present, *verbal* feedback, which is not then transferred to the assessment forms, may also take place. A number of international institutions have implemented WBA smart-phone and tablet ‘apps’ which allow for real-time completion and uploading of the assessment feedback.

Another barrier to the provision of feedback in our study may have been the lack of an explicitly-titled free-text ‘*feedback*’ section; on these assessments the free text section was titled ‘*comments*’ and therefore was interpreted by some trainers as comments on the case, not on the trainee performance.

In our study, both at BST and HST level, trainees were more likely to complete DOPS assessments than the mini-CEX or CbD. This finding is in keeping with a UK study of dermatology trainees where the authors reported that 138 trainees completed 251 DOPS compared with 142 mini-CEX assessments (Cohen et al. 2009). In this study respondents reported that the Mini-CEX and Multisource Feedback (MSF) tended to feel more ‘artificial’ than DOPS; they also reported dissatisfaction with the quality of feedback provided on all assessments, despite an overall positivity about the benefits of WBAs. While there is limited empirical research exploring trainer and trainee preferences regarding assessment, it may be that trainers and trainees perceive DOPS as a more objective measure of performance as opposed to the more subjectively-perceived assessments of, for example, communication and professionalism. However, it is interesting to note that in a 2009 study of psychiatry trainees—for whom procedure-based WBAs are not usually required—Menon et al. (2009) also reported that trainees were ‘unimpressed’ with the introduction of these assessments, querying their reliability, validity and impact on the quality of training.

Our study found that the majority of WBAs took place in the second half of the year. This pattern, along with the limited provision of written feedback and follow-up assessments, appears to point towards a limited use of these assessments to inform learning and development. During the implementation of WBA in the UK, one 2011 study of paediatric trainees (Bindal et al. 2011) reported that WBAs were still viewed as a ‘tick-box’ exercise. Menon et al. (2012) reported that psychiatry trainers and trainees (Menon et al. 2009) understood that the introduction of WBA was both driven by a desire to improve training but that it was also ‘politically driven’; comments from these trainees also referenced the ‘tick-box exercise’ designed purely to fulfil end-of-year assessment requirements. In a recent review of the issues underlying the problems encountered in WBA implementation Swayamprakasam et al. (2014) also pointed towards the need for widespread communication strategies to inform—or re-inform—the understanding of the purpose of WBA.

The potential ‘floor’ and ‘ceiling’ effect of WBA also warrants further investigation. In this study, the low number of assessments documenting a competence that was ‘borderline’ or ‘below expectation’ raises a number of issues around ‘failure to fail’. The reluctance and anxiety of trainers around the delivery of negative feedback is well documented (Kogan et al. 2012) as are issues with the rating systems used to structure this feedback (Hassell et al. 1035). In our assessments, the use of an ‘expectations’ rating system (i.e. ‘above expectation’, ‘meets expectations’) in Mini-CEX and CbD assessments, without explicit reference to curriculum outcomes or competencies, may also have been perceived as overly-subjective and less conducive to learning.

This is the first large-scale study of WBA implementation in Ireland. The methodology employed to conduct the study was rigorous and quality checks were implemented to ensure the quality and accuracy of the data. The study provides and overview of the varied integration of the assessments since the introduction of the tools and has highlighted similar issues to those identified internationally. The study was designed to provide a thorough background in developing an extensive programme of research on WBA in the Irish postgraduate medical education context and will form the basis of a large in-depth qualitative study to explore the value of WBA to both trainers and trainees. The findings have also highlighted a number of areas for further development of the assessment, particularly regarding the implementation and assessment of same. One of the main limitations of the study lies in the evaluation of the quality of feedback; only written feedback was extracted which may not accurately or fully reflect the quality or richness of verbal feedback provided at the end of the workplace-based assessments.

## Conclusion

This study was developed as a ‘scene-setting’ exploration of what has happened within our medical training programmes at our institution since the introduction of workplace-based assessment in 2010; however it reflects and adds to the international body of work on workplace-based assessment implementation. As is the case internationally, issues persist in the successful implementation of formative assessment in postgraduate medical education. Recommendations based on this study and a subsequent larger qualitative study, are currently in motion with the aim of further contributing to the international discussion on the value of formative assessment in trainee development.

## References

[CR1] Ali J (2014). Workplace-based assessments: Lost in translation?. Clin Teach.

[CR2] Bennett RE (2011). Formative assessment: a critical review. Assess Educ Princ Policy Pract.

[CR3] Bindal T, Wall D, Goodyear HM (2011). Trainee doctors’ views on workplace-based assessments: Are they just a tick box exercise?. Med Teach.

[CR4] Black D, Welch J (2009). The under-performing trainee—concerns and challenges for medical educators. Clin Teach.

[CR5] Bok H, Teunissen P, Favier R, Rietbroek N, Theyse L, Brommer H (2013). Programmatic assessment of competency-based workplace learning: when theory meets practice. BMC Med Educ.

[CR6] Bok H, Teunissen P, Spruijt A, Fokkema J, Van Beukelen P, Jaarsma D (2013). Clarifying students’ feedback-seeking behaviour in clinical clerkships. Med Educ.

[CR7] Bok HGJ, Jaarsma DADC, Spruijt A, Van Beukelen P, Van Der Vleuten CPM, Teunissen PW (2015). Feedback-giving behaviour in performance evaluations during clinical clerkships. Med Teach.

[CR8] Clark I (2012). Formative assessment: assessment is for self-regulated learning. Educ Psychol Rev.

[CR9] Cohen S, Farrant P, Taibjee S (2009). Assessing the assessments: U.K. dermatology trainees’ views of the workplace assessment tools. Br J Dermatol.

[CR10] Cook D, Zendejas B, Hamstra S, Hatala R, Brydges R (2014). What counts as validity evidence? Examples and prevalence in a systematic review of simulation-based assessment. Adv Health Sci Educ.

[CR11] Cook DA, Brydges R, Ginsburg S, Hatala R (2015). A contemporary approach to validity arguments: a practical guide to Kane’s framework. Med Educ.

[CR12] Driessen E, Scheele F (2013). What is wrong with assessment in postgraduate training? Lessons from clinical practice and educational research. Med Teach.

[CR13] Fokkema J, Teunissen PW (2013). Assessing the assessment of interventions: we’re not there yet. Med Educ.

[CR14] Fokkema JPI, Teunissen PW, Westerman M, van der Lee N, van der Vleuten CPM, Scherpbier AJJA (2013). Exploration of perceived effects of innovations in postgraduate medical education. Med Educ.

[CR15] Hassell A, Bullock A, Whitehouse A, Wood L, Jones P, Wall D (1035). Effect of rating scales on scores given to junior doctors in multi-source feedback. Postgrad Med J.

[CR16] Hatala R, Cook D, Brydges R, Hawkins R (2015). Constructing a validity argument for the objective structured assessment of technical skills (OSATS): a systematic review of validity evidence. Adv Health Sci Educ.

[CR17] Kogan JR, Holmboe ES, Hauer KE (2009). Tools for direct observation and assessment of clinical skills of medical trainees. JAMA.

[CR18] Kogan JR, Conforti LN, Bernabeo EC, Durning SJ, Hauer KE, Holmboe ES (2012). Faculty staff perceptions of feedback to residents after direct observation of clinical skills. Med Educ.

[CR19] Menon S, Winston M, Sullivan G (2009). Workplace-based assessment: survey of psychiatric trainees in Wales. Psychiatr Bull.

[CR20] Menon S, Winston M, Sullivan G (2012). Workplace based assessment: attitudes and perceptions among consultant trainers and comparison with those of trainees. Psychiatrist.

[CR21] Nicol DJ, Macfarlane-Dick D (2006). Formative assessment and self-regulated learning: a model and seven principles of good feedback practice. Stud High Educ.

[CR22] Norcini JJ, Blank LL, Arnold GK, Kimball HR (1995). The Mini-CEX (clinical evaluation exercise): a preliminary investigation. Ann Intern Med.

[CR23] St-Onge C, Young M (2015). Evolving conceptualisations of validity: impact on the process and outcome of assessment. Med Educ.

[CR24] Swayamprakasam AP, Segaran A, Allery L (2014). Work-based assessments: making the transition from participation to engagement. JRSM Open.

[CR25] Vandenbroucke JP, Ev Elm, Altman DG, Gøtzsche PC, Mulrow CD, Pocock SJ (2007). Strengthening the reporting of observational studies in epidemiology (STROBE): explanation and elaboration. Ann Intern Med.

[CR26] von Elm E, Altman DG, Egger M, Pocock SJ, Gøtzsche PC, Vandenbroucke JP (2007). The strengthening the reporting of observational studies in epidemiology (STROBE) statement: guidelines for reporting observational studies. Prev Med.

[CR27] Watling C (2014). Cognition, culture, and credibility: deconstructing feedback in medical education. Perspect Med Educ.

[CR28] Watling C, Driessen E, van der Vleuten CPM, Vanstone M, Lingard L (2012). Understanding responses to feedback: the potential and limitations of regulatory focus theory. Med Educ.

[CR29] Watling C, Driessen E, van der Vleuten CPM, Vanstone M, Lingard L (2013). Beyond individualism: professional culture and its influence on feedback. Med Educ.

[CR30] Watling C, Driessen E, van der Vleuten CPM, Vanstone M, Lingard L (2013). Music lessons: revealing medicine’s learning culture through a comparison with that of music. Med Educ.

